# Livelihood Experiences and Adherence to HIV Antiretroviral Therapy among Participants in a Food Assistance Pilot in Bolivia: A Qualitative Study

**DOI:** 10.1371/journal.pone.0061935

**Published:** 2013-04-16

**Authors:** Kartika Palar, Alexis Martin, Martha Lidia Oropeza Camacho, Kathryn Pitkin Derose

**Affiliations:** 1 Pardee RAND Graduate School, RAND Corporation, Santa Monica, California, United States of America; 2 Fielding School of Public Health, University of California Los Angeles, Los Angeles, California, United States of America; 3 The World Food Program, Regional Office for Latin America and the Caribbean, Panama City, Panama; 4 The World Food Program, Bolivia Country Office, La Paz, Bolivia; 5 RAND Corporation, Santa Monica, California, United States of America; Partners for Applied Social Sciences (PASS), Belgium

## Abstract

**Introduction:**

Health and development organizations increasingly promote livelihood interventions to improve health and economic outcomes for people living with HIV (PLHIV) receiving treatment with antiretroviral therapy (ART). In-depth understanding about how PLHIV make labor decisions in the context of treatment for HIV – and treatment decisions in the context of their livelihoods – is essential to guiding intervention design and developing hypotheses for future research on livelihoods and ART. However, few studies have explored the perspectives of PLHIV regarding integration of livelihoods and ART in urban, resource-limited settings.

**Methods:**

Qualitative interviews explored the livelihood experiences of food insecure ART patients in four Bolivian cities (n = 211). Topics included work-related barriers to ART adherence, HIV-related barriers to work, and economic coping mechanisms. Themes were identified using content coding procedures, with two coders to maximize reliability.

**Results:**

Participants reported complex economic lives often characterized by multiple economic activities, including both formal and informal labor. They struggled to manage ART treatment and livelihoods simultaneously, and faced a range of interpersonal and structural barriers. In particular, lack of HIV status disclosure, stigma, and discrimination were highly salient issues for study participants and likely to be unique to people with HIV, leading to conflict around requesting time off for clinic visits, resentment from co-workers about time off, and difficulties adhering to medication schedules. In addition, health system issues such as limited clinic hours or drug shortages exacerbated the struggle to balance economic activities with HIV treatment adherence.

**Conclusions:**

Improved policy-level efforts to enforce existing anti-discrimination laws, reduce HIV-related stigma, and expand health services accessibility could mitigate many of the barriers discussed by our participants, improve adherence, and reduce the need for livelihoods interventions.

## Introduction

Livelihood programs and policies are increasingly promoted to support the economic well-being and food security of people living with HIV (PLHIV), improve antiretroviral treatment (ART) adherence and outcomes, and serve as a sustainable transition from food assistance in HIV treatment settings [1], [2], [3]. However, an in-depth understanding of how food insecure PLHIV experience their own livelihoods in relation to HIV treatment is still needed to inform appropriate interventions and policies for this group. Our study sought to address this gap in knowledge through a qualitative exploration of the perceptions of PLHIV about the trade-offs between ART adherence and labour decisions.

While studies suggest that health improvements accompanying ART may lead to renewed productive capacity and increased labour supply in resource-limited settings [4], the multidimensional ways in which people on ART experience their livelihoods, and how these in turn affect their treatment decisions, are not well-understood, particularly for urban settings. Few studies in low-income countries explore in-depth how PLHIV co-manage ART and work, the barriers they face in the quest to integrate their economic lives with the expectation of lifetime treatment, and how to use this information to develop livelihoods interventions in the context of ART.

Livelihoods can be defined as the “the capabilities, assets (including both material and social resources) and activities required for a means of living” [5], while food security can be defined as “physical and economic access to adequate food for all household members, without risk of losing such access” [6]. Proponents of a livelihoods approach to supporting food security prioritize the long-term well-being of PLHIV along multiple dimensions, including economic productivity, health, and sustained access to food and nutrition.

Throughout Latin America and the Caribbean (LAC), widespread inequalities, discrimination and poverty are significant factors shaping the HIV epidemic [7]. Concentrations of extreme poverty in rural areas fuel seasonal labour migration and attendant HIV risks, and have also contributed to rapid urbanization and increasing numbers of people living in extreme poverty in cities where conditions are ripe for the rapid spread of HIV [8]. While LAC governments have made strong progress towards universal access to ART over the last decade, access to comprehensive HIV care – which includes attention to food and livelihood security – remains low in resource-limited settings throughout the region [9], [10], [11].

In response to policy gaps addressing nutrition and food security for PLHIV in LAC, WFP's Regional Office for Latin America and the Caribbean (WFP-LAC) began in 2008 to pursue strategies to support regional governments' capacity to integrate food and nutritional interventions with HIV treatment and care, including attention to livelihoods. Recognizing that food security interventions for PLHIV must support sustainable health outcomes, WFP also encourages food-based interventions worldwide to incorporate livelihoods strategies that contribute to the long-term food security, nutritional recovery and ART adherence of its beneficiaries. However, WFP's food-based interventions for PLHIV are relatively new in Latin America, and livelihoods interventions have yet to be comprehensively developed and implemented.

We thus set out to investigate the qualitative livelihood experiences of food insecure ART patients in Bolivia who were part of a clinic-based pilot project offering food assistance and nutritional education, sponsored by the WFP-LAC. The food and nutrition-based pilot did not include a structured livelihoods component at its inception, although the WFP was interested in adding one in the future. We explored the current work, economic, and HIV treatment experiences of food pilot participants in order to inform the creation of future livelihoods interventions for food insecure people living with HIV, and to contribute to knowledge about how livelihoods and ART adherence are related in an urban, Latin American setting.

## Methods

### Background of Research Collaboration

This study involved collaboration between the WFP-LAC and the RAND Corporation, a nonprofit research organization based in the United States. In 2008–2009, WFP/RAND began implementing joint activities in Bolivia by conducting qualitative, formative research on the dietary habits and nutritional status of people living with HIV receiving ART. The data from this research were used to design pilot food assistance and nutritional education interventions for people with HIV in Bolivia during 2010 and 2011.

### Ethics Statement

Ethical approval for the livelihoods study was obtained from RAND's Human Subjects Protection Committee. Study materials were also approved by the Bolivian national institutional review board, Comité Nacional de Bioética, Comisión de Ética de la Investigación (CEI). In addition, the WFP-LAC, WFP-Bolivia, and members of the Bolivian National AIDS Program reviewed and approved all study materials. Participation in the study was completely voluntary and not a condition for receiving food assistance. Informed written consent was obtained from all participants.

### Data Collection

WFP/RAND implemented a study of livelihood and economic coping experiences with a sample of food insecure ART patients in Bolivia participating in the food assistance pilot sponsored by WFP-LAC. Four clinics participated in the WFP food pilot, one clinic each in the cities of La Paz, El Alto, Cochabamba, and Santa Cruz, which were selected to represent the diverse urban populations of Bolivia. La Paz – the capital city – and El Alto form one large metropolitan area in the mountainous and relatively cold Andean region. El Alto was previously a suburb of La Paz populated primarily by indigenous migrants from the Andean highlands and is now its own city almost equal in size to La Paz. Cochabamba is located in the hill region between the Andes and the Amazon basin, and is the smallest and most temperate of the study cities. Santa Cruz is located in lowland tropical plains on the edge of the Amazonian basin and is both the largest and most commercial city in our study.

The study sampling frame was the universal set of participants recruited into the food assistance pilot at these four clinics during its first six weeks (November – December 2010). Inclusion criteria for both the food pilot and the study were being on ART and having household food insecurity. Exclusion criteria for participation in the study were being hospitalized or bedridden due to illness, not speaking Spanish, or being under age 18. Participation in the pilot or the study was not tied to ART adherence.

Face-to-face interviews consisted of a semi-structured qualitative interview and a closed-ended questionnaire (n = 211), which together took approximately one hour. The qualitative interview was the focal point of the study, used to gather data on the perceptions of PLHIV about how they integrate ART and livelihoods (i.e. our primary study question). The closed-ended questionnaire was used principally to collect standardized data on key demographic and socioeconomic characteristics that would be useful in describing the study population. The entire interview was administered orally, to enable patients of all literacy levels to participate. The study protocols were developed in English and professionally translated into Spanish. Members of Asociación Un Nuevo Camino (ASUNCAMI), part of Bolivia's national network of PLHIV (or REDBOL), reviewed the protocols for language and cultural appropriateness in the Bolivian context and participated in pilot testing, which led to subsequent revisions and a final version of the instrument.

Qualitative research is especially suited to gather in-depth and nuanced information about human experiences, and is intended to capture the range and salience of experiences and phenomena. The qualitative interview explored livelihood experiences in depth, including the range of economic activities performed by participants in general and in the last week, how HIV treatment affected their livelihoods, and how their livelihoods affected HIV treatment adherence. Within the broad protocol topics, interviewers used probes and clarifying questions to draw out richness within participant stories. In addition, a closed-ended questionnaire was implemented to document characteristics of the study population, but was not the primary data source for analysis. This questionnaire was adapted from a Spanish-language version previously developed, validated and used by WFP/RAND in Bolivia and Honduras. It included questions on demographics, household composition, and socio-economic situation, including food insecurity (measured by the Latin American and Caribbean Food Security Scale or ELCSA)[12], and work status. Work status was measured using a series of questions asking if the participant 1) had worked in the last 6 months, 2) had worked in the last month, and 3) was currently working. Change in work situation since HIV diagnosis was also measured.

The study field team consisted of two lead researchers from the United States, both fluent in Spanish and with substantial experience in Latin America and in HIV, a Bolivian project manager, and three Bolivian interviewers from each of the three regions represented in our study. The Bolivian members of the research team all had extensive experience working with PLHIV as well as research-based interviewing. All stages of the research process, including the interviews, were conducted in Spanish.

Upon the advice of local partners, RAND provided a small monetary incentive of 10 Bolivianos (∼$1.75) to each respondent as a token of appreciation.

### Analysis

The interviews were transcribed in Spanish and analyzed in Spanish. We conducted extensive coding of interview transcripts to identify prominent themes using Atlas.ti, a qualitative text management software, performed by two coders (KP and AM) to maximize validity and reliability [13]. As a first step, we developed a codebook of overarching themes [14] based on the major topics in our interview protocol. Second, we used content coding procedures to identify the presence of these themes [14], [15], [16] in combination with inductive approaches to identify new themes that we then added to the codebook [13], [17]. Transcripts were double coded at pre-determined intervals (every 20 transcripts) to facilitate a high level of agreement between coders. After coding was completed, we produced a summary of coding issues and analytic insights for each set of codes, and validated the independent work of the other coder. The Bolivian members of the research team reviewed quotes used in the paper for accuracy in capturing local meaning. Bilingual members of the research team translated the selected quotes from Spanish to English.

Data from the closed ended questionnaires were analyzed descriptively using univariate statistics for the study population as a whole, and between sub-groups (gender, study site). Sample characteristics were described using percentages or means. Differences between groups were tested using the Mann-Whitney or Kruskl Wallis test (depending on number of levels of the subgroup) for continuous variables, and Chi-squared tests (or Fisher's exact test where expected cell sizes were small) for categorical variables. Finally, the interview transcript data were summarized and arrayed into data display matrices (Ryan and Bernard 2000, 2003) side by side with data from the closed ended questionnaires to identify patterns and salience of themes across different types of respondents.

## Results

### Demographic and socio-economic characteristics

Table 1 summarizes the demographic and socio-economic characteristics of the study population from the closed-ended questionnaire. Almost half of all participants reported not working in the past month (41%). HIV diagnosis was reported to have resulted in a change of work status (49%). Women were less likely to have completed primary school (54% vs. 84%, p<0.01) and to have worked in the last month (51% vs. 73%, p<0.01) compared to men. Half of all participant households were severely food insecure, affecting a higher proportion of women than men (58% vs. 32%, p<0.01) and translating into a relative risk of 1.81 for women compared to men. Study sites differed significantly in the self-reported ethnicity of participants, reflecting regional differences. More than half of participants in La Paz and El Alto identified as indigenous (primarily Aymara), while only 20% of participants in Cochabamba (Quechua and Ayamara) – and only one person in Santa Cruz (Guarani) – identified as indigenous. No other participant characteristics presented in this study differed significantly by study site.

**Table 1:** Demographic and socio-economic characteristics of study population (n = 211).

	All(n = 211)	Women(n = 137)	Men(n = 74)	La Paz(n = 52)	El Alto(n = 32)	Santa Cruz(n = 62)	Cochabamba(n = 65)
Female, %	65	--	--	62	69	66	65
Indigenous, %^a^	28	29	26	**50**	**57**	**2**	**22**
Age group, %							
18–24	15	9	18	12	19	6	25
25–44	65	67	64	65	62	76	57
45–64	19	21	16	21	16	18	18
65+	1	1	1	2	3	0	0
Households with children < age 18, %	73	**80**	**61**	79	75	73	68
Mean household size^b^, n [SD]	3.2 [1.84]	**3.4 [1.9]**	**2.9 [1.7]**	3.8[2.3]	3.5[2.2]	3.0[1.7]	2.8[1.1]
Primary school or more, %	64	**54**	**84**	73	63	58	65
Worked in the last month, %	59	**51**	**73**	62	53	64	54
Work changed as result of HIV diagnosis, %	49	50	46	48	47	43	55
Severe food insecurity, %	49	**58**	**32**	43	44	63	42

Notes: Values in **bold** indicate a statistically significant difference (p<0.05) between genders or study sites in the row characteristic.

a Indigenous groups in La Paz and El Alto were Aymara (n = 20 and  = 15, respectively), followed by Quechua (n = 4 and  = 2, respectively). One person in La Paz and one person in Santa Cruz identified as Guarani. Primary indigenous groups in Cochabamba were Aymara (n = 5) and Quechua (n = 9). Those not identifying as one of the above indigenous groups indicated “*mestizo*” as their ethnicity. ^b^Household size excludes participant.

When asked to self-define their occupation in the closed-ended questionnaire, participants reported a range of livelihoods, which appeared to encompass both paid and unpaid work (though compensation was not explicitly referred to in the question) (Table 2). Top occupations reported by women included being a “housewife” (36%), commercial enterprise (18%), and services (16%), such as domestic employee, sewing, washing clothes, or childcare. Top occupations for men also included services (16%), although of different types than women, such as food and drink service, gardening, and transportation. Manual labor was the next most common occupation reported by men (14%).

**Table 2:** Top occupations of study participants, % (n).

	All	Women	Men
Housework^1^	26%	36%	8%
Services^2^	16%	16%	16%
Commerce^3^	14%	18%	8%
Manual labour^4^	6%	2%	14%
Health^5^	4%	4%	5%
Education	3%	1%	5%
Arts/Entertainment^6^	2%	1%	4%
Industry/Manufacturing^7^	2%	0%	5%

1 Refers to housework for one's own household, not domestic household work for others. Women tended to report this occupation as being an “*ama de casa*”, or housewife. Men tended to report this occupation as “*labores de casa*”, or housework.

2 Includes work as a domestic employee, food and drink service, gardening, sewing, washing clothes, childcare, transportation, etc.

3 Includes both entrepreneurial or employer-based commerce.

4 Includes construction, recycling, laborer-for-hire, etc.

5 Includes medical/nursing positions, as well as being an HIV peer counselor.

6 Includes artisans, painters, and actors.

7 Includes industrial mechanic, garment manufacturer, factory worker, etc.

### Economic activities comprising livelihoods

In the qualitative interviews, participants described more frequent and complex economic activity than they indicated in the closed-ended questionnaire. While discussing their activities in the last week, almost all participants discussed engaging in at least one economic activity during the last 7 days, in contrast to results from the closed-ended questionnaires, which suggested half of the sample had not worked in the last month. Qualitative analysis revealed that one third of participants – roughly the same proportions for men and women - reported taking on two or more economic activities in the last week. However, a much higher proportion of women than men reported taking on more than three economic activities. Notably, this trend was similar among women reporting “housewife” as their occupation in the closed-ended questionnaire, indicating that being a housewife did not preclude economic activity. Women were much more likely than men to report piecing together various small work opportunities in order to earn income. For example, one woman in La Paz described her overall set of economic activities:

Sometimes I wash clothes, or somebody has a small job for me that I know how to do, sometimes cooking…sometimes cleaning…it really depends on whatever people tell me they need.

Women also reported having specific people or businesses from the community they relied on for extra work for when times were tight, especially washing clothes or dishes. These arrangements were not always for cash – they often involved work in exchange for food, as one woman in Santa Cruz described:

If I see there's no food in the house, I go to help out at the market, to the food stand, to peel vegetables, wash dishes, and then they give me soup and I bring it home so my family can eat… I'm constantly trying to figure out where the food is coming from.

Notably, very few people talked about engaging in agriculture as part of their economic activities. Those who did mention agriculture would episodically travel outside of the city to family farms during peak planting and harvest times, rather than engage in urban farming. Similarly, very few people mentioned using home gardens as a strategy to augment food stores or alleviate food shortages.

### Dual management of treatment and livelihoods

Within the context of the livelihoods reported by our study participants, dual management of HIV treatment and livelihoods emerged as a salient theme. By “dual management” we refer to both how people managed their livelihoods in light of HIV treatment demands, and how people managed their ART treatment regimens given the structure of their livelihoods. Within dual management, we found two main sub-themes: negotiating time off from work and staying healthy at work, both strongly related to issues of disclosure of HIV status, and HIV-related stigma and discrimination.

#### Getting permission: “Time off for the doctor? You look fine!”

A common theme discussed by participants was the issue of managing work schedule and HIV status disclosure in the context of ART medication regimens and medical appointment schedules. Most people with outside employment reported that they had not disclosed their HIV status to their supervisors, nor to coworkers, with no notable difference in disclosure by gender or occupation. Fear of discrimination, particularly fear of being fired, was the most common reason given for non-disclosure; however, participants also reported internalized stigma, such as feelings of shame or perceptions of low self-worth, as an additional reason for non-disclosure.

Lack of HIV disclosure at work introduced internal or interpersonal conflict into the workplace for many participants when they had to take time off to attend doctor appointments or pick up medication. Time off from work could only be secured by asking ‘permission’, which was not always given. Meanwhile, lack of disclosure prevented the participant from explaining the importance of the request. Thus, asking for permission was often expressed as a strategic action – when to ask for it, how often, and what to do if permission was not given. In some cases, people invented other illnesses or reasons why a visit to the doctor was necessary; however, this sometimes prompted employers to demand doctor's notes or medical histories, forcing a choice between keeping their HIV status confidential (and risk losing their job from missing too much work) or disclosing (and risk losing their job because of discrimination). One man employed as a technician in La Paz talked about the consequences of refusing to share his medical history with his boss:

Of course, [my bosses] wanted to ask me for my medical records, saying that I go to the doctor all the time. They didn't want to give me permission to go. But I told my boss, ‘I don’t have to give you my medical records', because it's true. But now I'm in a bad situation at work because of this.

In addition to trouble with bosses, our participants also reported trouble with colleagues, including jealousy at the perceived benefit of additional time off. One woman in Cochabamba discussed having a difficult experience managing co-workers, bosses, and her treatment schedule:

At my work, they don't give me permission [to go to the doctor], and also there's a woman [coworker] who is always saying “why does she get so much time off, what's that about?” Everyone notices, everyone asks me why I go to the doctor, but I don't tell them anything. They say “You, what can you possibly have, since you look just fine?” It's difficult, having to explain. But even worse would be to tell them [about my HIV status] because I'd get fired in an instant.

Several people facing this situation chose jobs with less consistent hours and less stability in order avoid dealing with the constant threat of punishment or harassment for asking for too much ‘permission’. For example, one man in La Paz reported choosing to work as a day laborer, despite its inconsistency, in order to gain flexibility around his treatment schedule:

I do [day labor work] in order to come here [to the clinic], for my treatment. I don't look for a stable job, because then I wouldn't be able to be absent as much. I have to stay with the work I have, because it gives me more flexibility to come, pick up my medicines, do my lab tests, or whatever I need to do. In my jobs now, I can work when I choose, but in a regular job I can't – if I even miss one day they let me go and I'd be without work at all.

Given the difficulty of dual management of livelihoods and treatment, some people risked their livelihoods by choosing to skip work on days when they knew they had to attend the clinic. Still others risked poor adherence by deciding not to keep their appointments or not to pick up their meds at the allotted times in order to avoid problems at work.

#### Staying healthy at work: “My medications are the most important thing.”

Medication-taking behavior was also affected by workplace issues around disclosure for participants in our study. Since most people had not disclosed their HIV status to their bosses or co-workers, they had to either take ARVs in secret (e.g. the bathroom) or lie and say ARV medications were ‘vitamins’ or pills for a common illness. At times, the daily pill-taking was noted by employers and the person was asked to provide medical records to assure the employer that the employee was healthy and capable of working.

Very few people reported failing to take their pills at the prescribed time in order to avoid conflict at work – rather, people implemented creative ART adherence strategies to avoid being pressured to disclose or have their regimen detected. A few people did note that they were able to tweak their medication schedule, in consultation with their doctor, to facilitate pill-taking right before or right after work, in order to avoid taking medications at work entirely. One woman in El Alto who worked in childcare shared her experience of working through the day despite painful medication side effects:

I have side effects…nausea, headache, and there are times that my body hurts so much that it leaves me paralyzed for a while and I can't stand up quickly because it hurts my feet. But luckily I'm without supervision most of the time at my work, so no one notices anything. I just suffer through it, stay silent. They don't notice, and I don't tell anyone that I'm in pain.

Overwhelmingly, participants in our study reported taking extraordinary measures to maintain adherence and livelihoods despite the barriers they confronted in their work places.

### Structural barriers to livelihoods and adherence

#### Getting treated: “Sometimes I can't get my medicines”

Participants also reported difficulties with the dual management of livelihoods and treatment in relation to characteristics of the health care system. In particular, the limited schedules and geographic location of the clinics where participants received treatment posed significant barriers. Many participants noted that the clinics closed too early at night or opened too late in the morning for them to schedule doctor appointments or pick up medications without conflicting with work schedules. This included both people working as employees for others, who tended to have more fixed schedules, as well as those working for themselves, such as market sellers, who tended to have more flexible schedules. For the latter, although they didn't have the issue with asking for permission to leave work, their prime selling hours were often the same hours needed to attend the clinic – thus, taking time away to attend the clinic compromised their economic security. Finally, there was only one public HIV clinic in each city, the location of which was not always easily accessible by all study participants.

In addition, some people reported issues with limited clinic staffing and resources, which caused them to waste valuable work time without gaining the health benefits of treatment. For instance, participants noted that at times the pharmacy was unstaffed during open hours or was out of specific medications needed by the client. In these cases, they had to return another day, requiring yet another round of permission or absence from important livelihood activities, as described by one woman from Santa Cruz:

Sometimes when I come there aren't medicines…like the last two times I came there weren't medicines, and they didn't tell me before, so then I had to return another time, yet again.

#### Earning a living through work: “There's just no jobs…and prices keep rising”

Participants in our study also discussed broader economic barriers to livelihoods in Bolivia, such as lack of overall work opportunities, lack of specific work opportunities that are flexible to HIV-related needs, and difficulties negotiating market dynamics.

The lack of overall work opportunities and widespread unemployment were issues of great concern to many of our study participants. Even as they talked about the specific ways that HIV and ART affected their livelihoods, they also recognized lack of opportunities and unemployment, particularly for those with low skills or education – as systemic problems affecting people in their communities regardless of health status. Younger adults with less work experience, people with less education, women with young children and no child care, and older adults past prime working age reported particular difficulty finding work.

Appropriate work was even scarcer when HIV-related needs were taken into account. In particular, participants reported many problems maintaining jobs involving strenuous labor (including manual labor or service work such as washing clothes, etc.) due to illness episodes or ARV side effects. However, locating less strenuous work – such as office jobs – often required a level of education, qualifications, and personal connections that many of our participants lacked as a result of socio-economic disadvantage. Some participants also noted that while their current job did not always provide a good income, it was better than the uncertainty of finding a better job. One man in La Paz noted that while he'd like to find better, less strenuous work, searching for a better job would be destabilizing to his health:

“I think that looking for another job will be hard and could harm me – I'll feel more tired, I may forget my medications, I feel like it will do worse for me. ”

Study participants who worked primarily in entrepreneurial activities – e.g. artisans, vendors, etc. – also reported structural difficulties in earning enough income to sustain themselves and their families. Entrepreneurs reported having a hard time finding markets for their goods that were both lucrative and geographically accessible. Geographically accessible markets tended to have fierce competition, while geographically far markets has less competition but posed other challenges that affected ART schedules (e.g. ability to return home in time for meals, ability to attend the clinic, having a place to take medications privately, etc).

## Discussion

This study is one of the first to explore how food insecure people with HIV dually manage ART with their livelihoods in an urban, resource-limited context, and, to our knowledge, the first to explore this issue in Latin America. Our findings add to a growing body of literature exploring the relationship between work, adherence and stigma in resource-limited settings. In qualitative interviews study participants identified negotiating permission for time off to attend the clinic, as well as taking daily ARVs at work, as major ongoing sources of conflict with bosses or coworkers, to whom the participant had almost never disclosed their HIV status because of stigma and fear of discrimination (e.g. getting fired). To avoid conflict related to ART schedules in the context of non-disclosure and fear of termination, many participants in our study chose to trade-off more lucrative or more stable work for more flexible but lower paying and less stable jobs in order to accommodate health care needs.

A small number of studies examining adherence more generally have identified unemployment and fear of lost work time as barriers to adherence [18], [19]. However, little is known about how trade-offs between livelihoods and ART adherence are perceived by PLHIV in resource-limited settings. Fear of disclosure has been identified as a barrier to adherence in developing countries [20], [21], while discrimination in the workplace against people with HIV have also been widely documented [22], [23]. One study on ART adherence in Botswana found that among individuals citing ‘frequency of required clinic visits’ as a barrier to adherence, ‘can’t leave work' was the most commonly stated reason, although there was no further explanation of why the individual felt unable to leave [24]. Meanwhile, unwillingness to ask for permission from employers to attend clinic visits was identified as a barrier to adherence among outpatients in Benin [25].

Employment discrimination based on HIV status – including forced disclosure, exclusion within the workplace, and termination – is particularly severe in Latin America and the Caribbean [22]. National law in Bolivia prohibits HIV-related discrimination by employers, protecting against termination based on HIV status [26]. However, the fact that workplace stigma was such a pervasive concern among our participants and yet none mentioned the existence of legal protections suggests that these protections were either largely unknown, or have not been widely or visibly enforced. Policy makers may be able to improve livelihoods and treatment outcomes for PLHIV by better communicating and enforcing antidiscrimination laws, while advocacy organizations and NGOs can play an important role in promoting workplace, community, or national stigma reduction. Broader based stigma reduction efforts are important to all PLHIV, but particularly those who work in the informal sector and are unlikely to directly benefit from formal workplace interventions.

Insufficient economic resources also pose a barrier to ART adherence, such as lack of food, transport or housing [19], [27], [28]. These often coincide with livelihood insecurity [18], as well as structural poverty and inequality [8], which may disproportionately affect women. An important message from our study is that PLHIV often face multiple layers of disadvantage which must be recognized by programs seeking to implement food security and livelihoods interventions. The structural barriers cited by our study participants, such as lack of economic opportunities, overall unemployment, and rising prices, while not unique to PLHIV, were intensified by HIV-specific challenges such as reduced physical strength, medication schedules, stigma and discrimination, and costs related to treatment. Often HIV-related challenges were compounded by socioeconomic or structural challenges, and vice versa, suggesting that people receiving ART in resource limited settings require interventions that recognize and address these multiple layers of disadvantage in coordination, rather than in isolation.

Improvements in the structure of health care could thus play a key role in alleviating issues related to both livelihoods and ART access and adherence. Health care barriers stemming from limited clinic resources affected our participant's ability to get the care they needed even when they were able to get time off work, and sometimes intensified problems at work, affecting productivity and economic well-being. Expanding clinic schedules (including staffing), improving communication with patients, and developing initiatives to ensure sustainable supply chains of essential medicines, could help to address these problems. Previous research in sub-Saharan Africa has found that adapting patient appointments to their work schedules can reduce default from HIV care [29]; another study suggested a 24-hour clinic as one solution to improve patient access and adherence to ART [28]. Our results suggest that such changes to health facilities could not only have an important effect on adherence, but on the ability of PLHIV to manage their work schedules and relationships with employers vis-à-vis ART. Thus, from a livelihoods perspective, improving the human and financial resources available to overburdened health facilities remains an important policy goal.

Researchers have begun to describe and evaluate integrated health and livelihoods programs in the scientific literature, but much is still unknown with regards to optimal program and policy design [3], [30], [31], [32], [33]. Results from one study in Zambia and Kenya suggest that leveraging existing livelihood networks, providing skills training and facilitating asset accumulation are the most promising approaches to support the food security of people on ART [32]. Although the literature is sparse in all geographic areas, most existing data on HIV and livelihoods comes from rural settings in sub-Saharan Africa, which are characterized by a different set of livelihoods and food security issues than in urban settings [34] and in Latin America.

Our study has limitations. One drawback is that we were unable to interview people before they started receiving food assistance, so it is possible that the addition of the food basket to their households affected their reports of labor participation and work decisions several months later. However, studies on the impact of food assistance on labor supply in other settings are inconclusive as to whether a labor response exists, and in what direction it goes [35], [36]. In addition, we did not have reliable clinical data on objective health status, ART adherence, and time on ART for participants. Therefore, we were not able to characterize the ART adherence or health status of our participants, nor explore the roles that livelihoods play in adjusting to life on ART over time. It is likely that people in different stages of treatment may require different kinds of livelihood support or interventions, as they regain their health and adapt to living with HIV as a chronic condition. These should be priorities for future study. Nevertheless, as an exploratory study on a topic where little research has been done, our use of qualitative methods with a group of food insecure adults on ART was appropriate to illuminate the range of adherence and livelihood issues confronted by ART patients in resource-limited, urban settings. Finally, this study was not intended to be representative of food insecure people receiving ART in Bolivia. In particular, using a sample of participants in a food pilot may be prone to selection issues, depending on how different they are from other food-insecure ART patients. However, it is worth noting that the cities targeted for the food pilot were the areas hardest hit by HIV in Bolivia, that our study took place in the sole government-run HIV clinic in each urban area, that the food pilot included all ART patients assessed as food insecure, and that eligible individuals almost universally accepted to participate in both the food pilot and the study. Therefore, while not representative, we believe that our study is likely to capture the range livelihood and treatment experiences of food insecure people currently accessing ART in urban areas of Bolivia.

This study adds to the literature by describing how food insecure PLHIV in an urban Latin American context manage their livelihoods in the context of ART and vice versa, including the primary barriers they faced and their response to these barriers. It is essential that policy makers tasked with creating and enforcing workplace and social protection policies for PLHIV, as well as promoting ART access and adherence, understand how PLHIV manage their treatment with regards to their livelihoods and socioeconomic situations, the barriers they face, and the strategies they use to overcome them. Policy initiatives should address specific barriers identified by PLHIV and improve the opportunities they have to thrive in all aspects of their lives, including in their livelihoods. Comprehensive HIV care and support packages must integrate health, social and economic components that are supported by strong national HIV policy and link to national social protection and social safety net programs. In the highly unequal context of LAC [37], where the most vulnerable populations face severe economic and food insecurity, it is even more crucial that livelihoods be addressed in coordination with health and social programming.
